# The Gut-Liver Axis in Nonalcoholic Fatty Liver Disease: Association of Intestinal Permeability with Disease Severity and Treatment Outcomes

**DOI:** 10.1155/2022/4797453

**Published:** 2022-01-31

**Authors:** Yu-Pei Zhuang, Yi-Ting Zhang, Ruo-Xin Zhang, Hao-Jie Zhong, Xing-Xiang He

**Affiliations:** ^1^Department of Gastroenterology, The First Affiliated Hospital of Guangdong Pharmaceutical University, Research Center for Engineering Techniques of Microbiota-Targeted Therapies of Guangdong Province, Guangzhou, China; ^2^South China University of Technology, Guangzhou, China

## Abstract

**Objective:**

To investigate the association between intestinal permeability and severity of nonalcoholic fatty liver disease (NAFLD) and the value of intestinal permeability in predicting the efficacy of metabolic therapy for NAFLD.

**Methods:**

Disease severity was compared between patients with normal and elevated intestinal permeability; correlations between D-lactate and different NAFLD parameters were analyzed; and the effects of metabolic therapy on NAFLD patients with normal and elevated intestinal permeability were evaluated.

**Results:**

A total of 190 patients with NAFLD were enrolled. NAFLD patients with elevated intestinal permeability had significantly higher levels of liver test parameters, liver ultrasonographic fat attenuation parameter, triglyceride, homeostasis model assessment of insulin resistance value, and diamine oxidase (all P˂0.05) than NAFLD patients with normal intestinal permeability. Furthermore, serum D-lactate levels were positively correlated with alanine transaminase, aspartate transaminase, gamma-glutamyl transpeptidase, total bilirubin, indirect bilirubin, fat attenuation parameter, triglyceride, and diamine oxidase (all *P* ˂ 0.05). Moreover, NAFLD patients with elevated intestinal permeability showed less improvement in TG levels (*P* = 0.014) after metabolic therapy.

**Conclusion:**

Intestinal permeability correlates with the disease severity in patients with NAFLD. Moreover, intestinal permeability may have value for predicting the efficacy of metabolic therapy for NAFLD patients.

## 1. Introduction

Nonalcoholic fatty liver disease (NAFLD) has emerged as the major cause of chronic liver disease worldwide, and its global prevalence is estimated to be 25% [1, 2]. NAFLD encompasses a spectrum of pathological changes, ranging from steatosis and nonalcoholic steatohepatitis (NASH), to liver cirrhosis and even hepatocellular carcinoma [3]. Considerable progress has been made toward understanding the pathogenesis of NAFLD, including the contributions of insulin resistance, inflammation, and oxidative stress [4–6]; however, the factors contributing to disease severity and progression have not been completely clarified.

There is evidence for a close interaction between the gut and the liver, known as the “gut-liver axis” [7, 8], and gut microbiota, microbial metabolites, and immune responses are associated with NAFLD pathogenesis [8–10]. Furthermore, recent studies have suggested a role for intestinal barrier dysfunction in the progression of NAFLD [11–13]. In animal studies, increased intestinal permeability can be detected in mice with NAFLD induced by high-fat or choline-deficient diets [13, 14]. In addition, clinical studies have demonstrated increased intestinal permeability in patients with NAFLD relative to healthy controls [12, 13]. Moreover, a previous study reported a correlation between the lactulose/mannitol ratio and pathologic severity of NAFLD, indicating that intestinal permeability might correlate with the severity of NAFLD [12]. However, another investigation detected no significant differences in liver transaminases or triglycerides between NAFLD patients with normal and increased intestinal permeability [15]. Hence, further investigation is needed to fully understand whether intestinal permeability is associated with disease severity in NAFLD patients, especially with respect to liver test parameters and blood lipid levels. Furthermore, recent studies have shown that some therapies can ameliorate NAFLD by improving the gut barrier permeability, indicating a potential role for addressing intestinal permeability in NAFLD treatment approaches [16, 17]. Nonetheless, it is still not known whether the treatment effect of NAFLD is affected by intestinal permeability.

Polyethylene glycol, ^51^Cr-labelled ethylene diamine tetraacetate acid (^51^Cr-EDTA), and a number of noninvasive tests (i.e., urinary recovery of orally administered sugars) are widely used to measure intestinal permeability in humans [18]. More recently, D-lactate, which is only produced by intestinal bacteria, has been also introduced as a convenient and well-accepted biomarker for intestinal permeability [19, 20]. Given that this compound is found in small concentrations in human blood, the elevated level of serum D-lactate indicates increased intestinal permeability.

This study was designed to investigate the association between intestinal permeability and severity of NAFLD and the value of intestinal permeability for predicting the efficacy of metabolic therapy for NAFLD with the use of serum D-lactate.

## 2. Methods

### 2.1. Participants

This retrospective study was performed in accordance with the ethical guidelines of the Declaration of Helsinki and approved by the ethics committee of the First Affiliated Hospital of Guangdong Pharmaceutical University (no. 20176601). Informed consent was obtained from all patients. We followed the STROBE (Strengthening the Reporting of Observational Studies in Epidemiology) statement in reporting this study. The sample size was estimated using an online software (Power and Sample Size Calculators; HyLown Consulting LLC, Atlanta, GA, USA) based on patients' fat attenuation parameter (FAP).

Patients with NAFLD who were hospitalized at the First Affiliated Hospital of Guangdong Pharmaceutical University for metabolic therapy between October 2017 and October 2019 and tested for D-lactate were eligible for inclusion. Patients with carcinoma; severe heart, brain, or kidney disease; other chronic liver diseases such as viral, alcoholic, autoimmune, and Wilson's disease; and those with missing important medical data were excluded.

**Figure 1:**
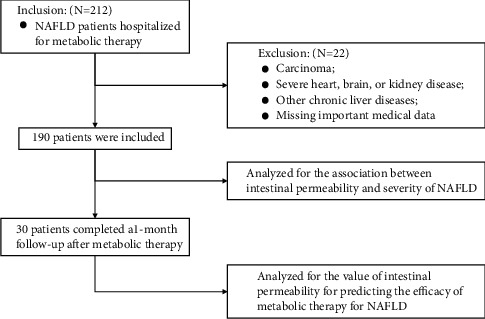
Selection process of patients with nonalcoholic fatty liver disease.

Data of all enrolled patients were used to analyze the association between intestinal permeability and severity of NAFLD. Data of those patients who completed a one-month follow-up after metabolic therapy were used to analyze the value of intestinal permeability for predicting the efficacy of metabolic therapy for NAFLD (Figure 1).

### 2.2. Data Collection

Medical records were reviewed, and the following information were extracted: demographic characteristics; smoking status; comorbidity; liver parameters (alanine transaminase (ALT), aspartate transaminase (AST), gamma-glutamyl transpeptidase (GGT), total bilirubin (TBIL), direct bilirubin (DBIL), and indirect bilirubin (IBIL)); metabolic parameters (triglyceride (TG), total cholesterol (TC), high-density lipoprotein (HDL), low-density lipoprotein (LDL), and homeostasis model assessment of insulin resistance (HOMA-IR) value); ultrasonographic parameters (liver stiffness measurement (LSM) and FAP); and intestinal parameters (serum D-lactate, diamine oxidase (DAO) and lipopolysaccharide (LPS)).

### 2.3. Definitions

NAFLD was defined as hepatic steatosis proven by imaging or histology, lack of secondary causes of hepatic fat accumulation, and no significant alcohol consumption (weekly alcohol intake ≤210 g in men and ≤140 g in women) [21]. Hypertension was defined by blood pressure ≥140/90 mmHg, use of antihypertensive medication, or a self-reported history of hypertension. Diabetes was defined by fasting glucose ≥7.0 mmol/L, 2h glucose ≥11.1 mmol/L during an oral glucose tolerance test, use of antidiabetic medication, or a self-reported history of diabetes. Elevated intestinal permeability was defined by elevated serum D-lactate (≥15 U/L). HOMA-IR value was calculated as previously described [22]. Metabolic therapy included lipid-lowering medicines (such as simvastatin and metformin) and lifestyle modifications (healthy eating and regular exercise).

### 2.4. Intestinal Mucosal Barrier Function Measurement

Blood samples were collected after patients had fasted for 8 h. The parameters of intestinal mucosal barrier function including D-lactate, DAO, and LPS were analyzed using the Intestinal Mucosal Barrier Biochemical Index Analysis System (JY-DLT, Beijing Zhongsheng Jinyu Diagnostic Technology Co., Ltd., China) according to the manufacturer's protocols [23, 24].

### 2.5. Statistical Analysis

All statistical analyses were conducted using SPSS 22.0 (IBM Corp., Armonk, NY, USA). Continuous variables are presented as mean ± standard deviation for those with normal distributions and as median and interquartile range for those with skewed distributions. Categorical variables are presented as numbers with percentages. Continuous variables were compared using the Student's *t*-test or Mann–Whitney *U*-test, and categorical variables were compared using the chi-squared or Fisher's exact tests, as applicable. Correlations between D-lactate and clinical parameters in patients with NAFLD were determined using Pearson's or Spearman's correlation, as applicable. *P* < 0.05 (two-sided) was considered to indicate statistical significance.

## 3. Results

### 3.1. Characteristics of Participants

We identified 190 eligible patients with NAFLD based on the inclusion and exclusion criteria. Most of the diagnoses of NAFLD (188/190) were made based on imaging, and only two patients were diagnosed with NAFLD by liver biopsy. Besides, 79(41.58%) had elevated intestinal permeability (D-lactate, ≥15 U/L) and 111 (58.42%) had normal intestinal permeability (D-lactate, <15 U/L). First, we compared the characteristics between these two groups of patients. There were no significant intergroup differences in demographic characteristics, smoking status, or comorbidities (Table 1).

### 3.2. Disease Severity in NAFLD Patients with Normal or Elevated Intestinal Permeability

Next, we analyzed disease severity in NAFLD patients with normal or elevated intestinal permeability. NAFLD patients with elevated intestinal permeability (D-lactate: 19.00 [16.48–23.23] U/L) had significantly higher levels of liver test parameters ALT, AST, GGT, TBIL, and IBIL (all *P* ˂ 0.05); higher levels of the liver ultrasonographic parameter, FAP (*P* = 0.001); higher levels of TG (*P* = 0.023) and an elevated HOMA-IR value (*P* = 0.020) among metabolic parameters; and a higher level of the intestinal parameter, DAO (*P* = 0.025), than those with normal intestinal permeability (D-lactate, 9.35 [6.47–12.40] U/L) (Table 2).

### 3.3. Correlation between D-Lactate and Disease Severity in Patients with NAFLD

Next, we assessed the correlations between D-lactate level and the clinical parameters identified as differing in NAFLD patients with and without elevated intestinal permeability. As shown in Table 3, D-lactate was positively correlated with ALT (*r* = 0.312, *P* ˂ 0.001); AST (*r* = 0.303, *P* ˂ 0.001); GGT (*r* = 0.190, *P* = 0.017); TBIL (*r* = 0.214, *P* = 0.004); IBIL (*r* = 0.247, *P* = 0.001); FAP (*r* = 0.252, *P* = 0.001); TG (*r* = 0.173, *P* = 0.021); and DAO (*r* = 0.218, *P* = 0.002). A positive correlation was also detected between D-lactate and LPS, although it was not statistically significant (*r* = 0.132, *P* = 0.069). However, D-lactate did not correlate with DBIL, LSM, TC, HDL, LDL, and HOMA-IR values (Table 3).

### 3.4. Efficacy of Metabolic Therapy in NAFLD Patients with Normal or Elevated Intestinal Permeability

Finally, we explored whether intestinal permeability can predict the efficacy of metabolic therapy in patients with NAFLD. Thirty patients with NAFLD completed a one-month follow-up after metabolic therapy. The effects of metabolic therapy were assessed by determining the improvements in blood lipids as follows: Δlipid = baseline levels – levels one month after treatment.

NAFLD patients with elevated intestinal permeability had a lower ΔTG value one month after metabolic therapy, at −0.10 (−0.39–0.39) vs. 1.00 (0.90–1.30) (*P* = 0.014) than those with normal intestinal permeability. Besides, patients with normal intestinal permeability seemed to have a better improvement of TC, HDL, and LDL after one month of metabolic therapy, although the differences were not statistically significant (Table 4). However, the changes in clinical characteristics (BMI); liver test parameters (ALT, AST, GGT, TBIL, DBIL, and IBIL); and liver ultrasonographic parameters (LSM and FAP) showed no significant differences between the two groups after one month of metabolic therapy.

## 4. Discussion

Our study showed that NAFLD patients with elevated intestinal permeability have more severe disease status, manifested as more serious liver dysfunction, hyperlipidemia, liver fat deposition, insulin resistance, and intestinal barrier damage. Our data also showed that serum D-lactate is positively correlated with parameters indicative of disease severity, including ALT, AST, GGT, TBIL, IBIL, FAP, and TG. These findings reveal a clear association between intestinal permeability and disease severity in patients with NAFLD, which add to accumulating evidence supporting the “gut-liver axis.” Hence, intestinal manifestations warrant increased attention in patients with severe NAFLD such as NASH and NAFLD-associated cirrhosis.

However, a previous study with a small sample size (35 NAFLD patients and 24 controls) reported no significant differences in liver transaminases and TG between NAFLD patients with normal and increased intestinal permeability, measured by ^51^Cr-EDTA excretion testing [15]; while another study reported a correlation between intestinal permeability and pathologic severity (portal inflammation, fibrosis, and ballooning of hepatocytes) using the lactulose-mannitol bowel permeability test, in line with our findings [12]. Furthermore, zonulin, a moderator of intestinal permeability, was found to be positively correlated with some parameters of disease severity, such as ALT, TG, HOMA-IR, and liver histopathology in patients with NAFLD, hence indicating a correlation between intestinal permeability and NAFLD severity [25]. In addition to the small sample size of the previous study being likely inadequate to show the relationship between intestinal permeability and disease severity in patients with NAFLD, using different methods to detect intestinal permeability may also lead to inconsistent results. In addition to ^51^Cr-EDTA excretion testing and lactulose-mannitol bowel permeability test, testing for D-lactate levels has become a widely used method for intestinal permeability detection in recent years [23, 24]. Owing to the simplicity of this method, the bias caused by multiple operations can be reduced. However, given that the criteria for defining intestinal permeability have not reached certain consensus, other tests to evaluate intestinal permeability should be used to confirm these findings by further studies.

There are two plausible reasons for the observed association between intestinal permeability and severity of NAFLD. First, elevated intestinal permeability can cause pathogenesis and progression of NAFLD. Animal research has shown that increased intestinal permeability induced by dextran sulfate sodium (DSS) enhances high-fat diet-induced hepatic inflammation and steatosis in mice [26]. Subsequent to the increase of intestinal permeability, bacterial components, particularly LPS, can translocate into the portal vein and thus the liver, resulting in liver inflammation and injury [27, 28]. In support of this hypothesis, a clinical study showed that plasma antibodies against LPS were increased in patients with NASH compared with healthy controls and increased with aggravated inflammation in NASH, indicating an association between LPS exposure and the severity of NASH in humans [29]. Consistent with this finding, our data showed a positive correlation between D-lactate and LPS, although the difference did not reach the threshold for statistical significance (*P* = 0.069), suggesting that increased intestinal permeability may lead to LPS translocation and consequently enhanced liver injury. The alternative explanation is that NAFLD may contribute to the increase in intestinal permeability, as disruption of the intestinal epithelial barrier and gut vascular barrier can be detected in NAFLD mice induced by high-fat diet [14].

Although NAFLD patients with elevated intestinal permeability had a higher level of DAO compared with those with normal intestinal permeability, the level of LPS was not significantly different between two groups. There are three possible explanations for this result. First, some potential confounders such as current alcohol consumption, which may affect the level of LPS in patients with NAFLD [30], were not considered. Second, genetic factors may influence the level of LSP through modulating intestinal permeability [31]. However, the data were missing in our study. Third, the small sample size may also contribute to the finding of no statistical difference between two groups.

Recent studies have found that improvement of intestinal permeability by fecal microbiota transplantation or probiotics had a therapeutic effect on NAFLD [16, 17]. These results suggest that improvement of intestinal permeability may promote the lipid-lowering effect of metabolic therapy in patients with NAFLD. Accordingly, our data revealed that NAFLD patients with elevated intestinal permeability present with less substantial improvement in TG levels after metabolic therapy. This study has provided evidence for the role of elevated intestinal permeability in NAFLD progression and suggested that a combination of treatment to improve intestinal barrier and metabolic therapy may have better therapeutic effects on NAFLD patients, especially those with elevated intestinal permeability.

However, we found that the improvement of clinical characteristics, liver test parameters, and liver ultrasonographic parameters were not significantly different between NAFLD patients with elevated and normal intestinal permeability after one month of metabolic treatment. As these patients received metabolic therapy, the improvement of blood lipids was more obvious, while other parameters of disease severity did not show significant improvement. In addition, we only analyzed the results of the one-month-long metabolic therapy; thus, the unexceptional improvement in other parameters could likely be attributed to the short treatment time. Therefore, a prospective and long-term follow-up study is needed to assess whether intestinal permeability affects the improvement of other parameters in patients with NAFLD after treatment.

The present study has some limitations. First, intestinal biopsies were not performed as standard in our investigation, as most enrolled patients had no gastrointestinal symptoms. Second, because this is a retrospective study, some potential confounders (e.g., use of medication with liver toxicity) that may lead to liver injury and steatosis were not recorded. Third, although our sample size was larger than those of previous studies investigating the association between intestinal permeability and NAFLD, this was a single-center study with a limited sample size. Only 30 patients' clinical data were available for analyzing the efficacy of metabolic therapy in NAFLD patients with normal or elevated intestinal permeability; moreover, we could not analyze whether intestinal permeability affects the improvement of other parameters in patients with NAFLD after treatment. Therefore, our results should be interpreted with caution, and future studies are needed to confirm these findings.

In summary, intestinal permeability correlates with the severity of liver dysfunction, hyperlipidemia, liver fat deposition, insulin resistance, and intestinal barrier damage in patients with NAFLD. Moreover, intestinal permeability may be valuable for predicting the efficacy of metabolic therapy in patients with NAFLD.

## Figures and Tables

**Table 1:** Patient characteristics.

	Normal intestinal permeability (*n* = 111)	Elevated intestinal permeability (*n* = 79)	*P* value
Age (years)	56.00 (48.00–64.00)	59.00 (50.00–65.00)	0.283
Male sex, *n* (%)	63 (56.76)	38 (48.10)	0.239
Body mass index (kg/m^2^)	25.71 ± 2.94	25.70 ± 3.53	0.990
Smoking, *n* (%)	28 (25.23)	19 (24.05)	0.853
Hypertension, *n* (%)	34 (30.63)	30 (37.97)	0.291
Diabetes, *n* (%)	18 (16.22)	18 (22.78)	0.255

Normal intestinal permeability was defined as D-lactate<15 U/L, and elevated intestinal permeability was defined as D-lactate≥15 U/L.

**Table 2:** Comparison of clinical parameters in NAFLD patients with normal or elevated intestinal permeability.

	Normal intestinal permeability (*N* = 111)	Elevated intestinal permeability (*N* = 79)	*P* value
*Liver test parameters*			
ALT (U/L)	18.00 (13.00–24.50)	24.00 (18.00–36.00)	˂0.001
	(*n* = 109)	(*n* = 75)	
AST (U/L)	19.00 (16.00–23.00)	21.00 (19.00–29.00)	˂0.001
	(*n* = 109)	(*n* = 75)	
GGT (U/L)	24.50 (18.00–32.53)	28.05 (20.95–45.50)	0.022
	(*n* = 92)	(*n* = 66)	
TBIL (*μ*mol/L)	11.00 (9.15–14.30)	12.90 (10.40–15.50)	0.010
	(*n* = 109)	(*n* = 75)	
DBIL (*μ*mol/L)	3.35 (2.70–4.30)	3.70 (2.80–4.80)	0.218
	(*n* = 108)	(*n* = 74)	
IBIL (*μ*mol/L)	7.80 (6.50–9.80)	9.20 (7.50–11.40)	0.003
	(*n* = 108)	(*n* = 75)	

*Liver ultrasonographic parameters*			
LSM (kPa)	6.70 (5.58–8.80)	6.80 (5.80–8.30)	0.924
	(*n* = 106)	(*n* = 76)	
FAP (dB/m)	259.50 (247.00–285.00)	276.00 (255.00–295.50)	0.001
	(*n* = 106)	(*n* = 77)	

*Metabolic parameters*			
TG (mmol/L)	1.37 (0.99–2.20)	1.68 (1.15–2.44)	0.023
	(*n* = 104)	(*n* = 73)	
TC (mmol/L)	4.70 (4.27–5.78)	4.98 (4.52–5.79)	0.096
	(*n* = 104)	(*n* = 73)	
HDL (mmol/L)	1.10 (0.96–1.30)	1.14 (1.00–1.25)	0.814
	(*n* = 104)	(*n* = 73)	
LDL (mmol/L)	2.82 (2.23–3.62)	3.04 (2.50–3.60)	0.258
	(*n* = 104)	(*n* = 73)	
HOMA-IR value	1.83 (0.74–2.62)	2.71 (1.14–3.91)	0.020
	(*n* = 110)	(*n* = 79)	

*Intestinal parameters*			
DAO (U/L)	4.54 (2.66–9.80)	6.15 (3.20–12.45)	0.025
LPS (U/L)	9.20 (4.39–10.51)	9.30 (4.18–11.22)	0.355

ALT, alanine transaminase; AST, aspartate transaminase; DAO, diamine oxidase; DBIL, direct bilirubin; FAP, fat attenuation parameter; GGT, gamma-glutamyl transpeptidase; HDL, high-density lipoprotein; HOMA-IR, homeostasis model assessment of insulin resistance; IBIL, indirect bilirubin; LDL, low-density lipoprotein; LPS, lipopolysaccharide; LSM, liver stiffness measurement; NAFLD, nonalcoholic fatty liver disease; TBIL, total bilirubin; TC, total cholesterol; TG, triglyceride. Normal intestinal permeability was defined as D-lactate<15 U/L, and elevated intestinal permeability was defined as D-lactate≥15 U/L. Significant *P* values are indicated in bold font.

**Table 3:** Correlations between D-lactate and clinical parameters in patients with NAFLD.

	D-lactate (U/L)	
	*r*	*P* value
*Liver test parameters*		
ALT (U/L)	0.312	˂0.001
AST (U/L)	0.303	˂0.001
GGT (U/L)	0.190	0.017
TBIL (*μ*mol/L)	0.214	0.004
DBIL (*μ*mol/L)	0.130	0.081
IBIL (*μ*mol/L)	0.247	0.001

*Liver ultrasonographic parameters*		
LSM (kPa)	0.049	0.515
FAP (dB/m)	0.252	0.001

*Metabolic parameters*		
TG (mmol/L)	0.173	0.021
TC (mmol/L)	0.117	0.121
HDL (mmol/L)	0.066	0.384
LDL (mmol/L)	0.096	0.203
HOMA-IR value	0.100	0.173

*Intestinal parameters*		
DAO (U/L)	0.218	0.002
LPS (U/L)	0.132	0.069

ALT, alanine transaminase; AST, aspartate transaminase; DAO, diamine oxidase; DBIL, direct bilirubin; FAP, fat attenuation parameter; GGT, gamma-glutamyl transpeptidase; HDL, high-density lipoprotein; HOMA-IR, homeostasis model assessment of insulin resistance; IBIL, indirect bilirubin; LDL, low-density lipoprotein; LPS, lipopolysaccharide; LSM, liver stiffness measurement; NAFLD, nonalcoholic fatty liver disease; TBIL, total bilirubin; TC, total cholesterol; TG, triglyceride. Significant *P* values are indicated in bold font.

**Table 4:** Effects of metabolic therapy in NAFLD patients with normal or elevated intestinal permeability.

	Normal intestinal permeability (*N* = 7)	Elevated intestinal permeability (*N* = 23)	*P* value
ΔTG (mmol/L)	1.00 (0.90–1.30)	−0.10 (−0.39–0.39)	0.014
Δtc (mmol/L)	0.09 (−0.12–0.55)	−0.20 (−0.81–0.49) (*n* = 22)	0.469
ΔHDL (mmol/L)	−0.02 ± 0.17	−0.03 ± 0.17	0.848
ΔLDL (mmol/L)	−0.04 (−0.37–0.00)	0.25 (−0.67–0.48)	0.598

Δ, baseline results minus results at one month after metabolic therapy; HDL, high-density lipoprotein; LDL, low-density lipoprotein; TC, total cholesterol; TG, triglyceride. Normal intestinal permeability was defined as D-lactate <15 U/L, and elevated intestinal permeability was defined as D-lactate ≥15 U/L. Significant *P* value is indicated in bold font.

## Data Availability

The data that support the findings of this study are available from the corresponding author upon reasonable request.

## Additional Points

Nonalcoholic fatty liver disease (NAFLD) has emerged as the major cause of chronic liver disease. Many intestinal factors (such as intestinal permeability) have been found to be associated with the pathogenesis, progression, and severity of liver diseases. The association between intestinal permeability and disease severity remains controversial. Intestinal permeability correlates with the disease severity in patients with NAFLD. Intestinal permeability may have value for predicting the efficacy of metabolic therapy for NAFLD patients.
